# Using Tri-Axial Accelerometry in Daily Elite Swim Training Practice

**DOI:** 10.3390/s17050990

**Published:** 2017-04-29

**Authors:** Sander Ganzevles, Rik Vullings, Peter Jan Beek, Hein Daanen, Martin Truijens

**Affiliations:** 1 Department of Human Movement Sciences, Faculty of Behavioural and Movement Sciences, Vrije Universiteit Amsterdam, Amsterdam Movement Sciences, 1081 BT Amsterdam, The Netherlands; p.j.beek@vu.nl (P.J.B.); h.a.m.daanen@vu.nl (H.D.); m.j.truijens@gmail.com (M.T.); 2 Faculty of Electrical Engineering, Eindhoven University of Technology, 5600 MB Eindhoven, The Netherlands; r.vullings@tue.nl

**Keywords:** training, monitoring, reliability, elite, compliance

## Abstract

*Background*: Coaches in elite swimming carefully design the training programs of their swimmers and are keen on achieving strict adherence to those programs by their athletes. At present, coaches usually monitor the compliance of their swimmers to the training program with a stopwatch. However, this measurement clearly limits the monitoring possibilities and is subject to human error. Therefore, the present study was designed to examine the reliability and practical usefulness of tri-axial accelerometers for monitoring lap time, stroke count and stroke rate in swimming. *Methods*: In the first part of the study, a 1200 m warm-up swimming routine was measured in 13 elite swimmers using tri-axial accelerometers and synchronized video recordings. Reliability was determined using the typical error of measurement (TEM) as well as a Bland-Altman analysis. In the second part, training compliance both within and between carefully prescribed training sessions was assessed in four swimmers in order to determine the practical usefulness of the adopted accelerometric approach. In these sessions, targets were set for lap time and stroke count by the coach. *Results*: The results indicated high reliability for lap time (TEM = 0.26 s, bias = 0.74 [0.56 0.91] with limits of agreement (LoA) from −1.20 [−1.50 −0.90] to 2.70 [2.40 3.00]), stroke count (TEM 0.73 strokes, bias = 0.46 [0.32 0.60] with LoA from −1.70 [−1.94 −1.46] to 2.60 [2.36 2.84]) and stroke rate (TEM 0.72 str∙min^−1^, bias = −0.13 [−0.20 −0.06] with LoA from −2.20 [−2.32 −2.08] to 1.90 [1.78 2.02]), while the results for the monitoring of training compliance demonstrated the practical usefulness of our approach in daily swimming training. *Conclusions*: The daily training of elite swimmers can be accurately and reliably monitored using tri-axial accelerometers. They provide the coach with more useful information to guide and control the training process than hand-clocked times.

## 1. Introduction

Coaches in elite sports carefully design individual training programs to optimize training effects in their athletes. A prerequisite for adequate evaluation of the effectiveness of the training process is the compliance of the athlete to the training schedule [1]. Compliance is the fulfillment of the training aims as defined by the coach. Traditionally, in swimming, coaches monitor training with stopwatches in order to determine the time over a specified distance, usually lap time and/or stroke rate [2]. Although simple, fast and practical in use, time recordings are subject to human error. Davey et al. [3] found a standard deviation (SD) of 0.6 s in manually timed 200 m freestyle trials compared to video-based timing. According to Hopkins [4] this would imply a typical error of measurement (TEM) of 0.42 s over this distance. In the 2016 Olympic 200 m freestyle for men this would have been the difference between the swimmer finishing in the second and the sixth place, respectively. This effect has also been observed in running, for which Ebben et al. [5] showed that manual timing was more prone to variability than electronic timing when measuring sprint times. Moreover, the error is likely to increase when the number of athletes supervised per coach increases. In swimming, coaches often simultaneously monitor 10 swimmers or more, starting, turning and finishing within short intervals from each other, sometimes even on different sides of the pool. This implies that coaches need to gain information on both lap time and stroke rate for all athletes in the pool at the same time. This highlights the need for more accurate monitoring tools in swimming, a need that has become even greater when realizing that the differences between elite athletes have decreased considerably over the years and increasingly larger investments are necessary to achieve performance gains [6]. For example, the relative difference between the first and last finisher in the men’s Olympic 100 m freestyle final has decreased gradually from 4.3% in 1976 to 1.7% in 2016.

When highly accurate and reliable measurements are required, coaches often use video with a standard frame rate of 50 Hz. However, while video analysis is very useful for accurate measurements and observational learning [7], it is too time-consuming for the monitoring of daily training practice, in particular because the aquatic environment causes considerable difficulties for the analysis of video footage [8,9]. Recent developments in water-resistant, commercially available tri-axial accelerometers provide opportunities for continuous monitoring of multiple swimmers with high reliability. Various studies have examined the use of accelerometers for the determination of lap time, stroke count and stroke rate for various stroke types [8,10,11,12,13]. For instance, Davey [11] showed that lap times derived from accelerometers were significantly more accurate than manually collected data. Several authors have demonstrated the validity and reliability of stroke rate and stroke count using either zero-crossing or peak detection algorithms [3,12,14,15,16]. Similarly, other relevant parameters can be detected such as kick count and rate [17]. In a recent review, Magalhaes et al. [18] concluded that accelerometers provide a reliable tool to assess the biomechanics of swimming performance. They suggested that they allow for continuous monitoring and for detecting changes in swimming technique due to fatigue on the basis of accelerometer output signals. Furthermore, there is wide methodological variation in the different studies concerning sensor location, swimming level and experimental protocol [18]. 

In brief, accelerometers constitute a promising tool for continuous monitoring of multiple athletes during training, but reports of their use during daily training of elite swimmers are lacking. Although good reliability and validity have been demonstrated [3,10,11,12,13,14,15,16,17,18,19,20], the experimental protocols used thus far were relatively short and did not cover whole training sessions. To our knowledge very few articles have studied all stroke types [12,15,16], and if so, this was typically done with a small number of subjects of undefined level [18] and without detection of the finish as described by Davey et al. [3]. In addition to higher reliability, accelerometers can be used to measure multiple parameters simultaneously enabling the coach to glean extra useful information from the measurements. It is particularly interesting to verify if swimmers fulfill the training aims as defined by the coach, i.e., if they show compliance [1].

Therefore, the present study was conducted to: (1) investigate the reliability and (2) the practical implementation of accelerometers in swimming training. The aims were to improve the algorithms from the current literature, for all swimming strokes, to measure a complete training session of elite swimmers, and to demonstrate application possibilities in the daily training environment. We hypothesized that: (1) stroke type, lap time, stroke count and stroke rate could be reliably measured for all stroke types for multiple swimmers simultaneously using tri-axial accelerometers, and (2) tri-axial accelerometers can be used to determine the compliance of elite swimmers to the training program prescribed by the coach, enabling fast and accurate feedback on multiple parameters simultaneously. The study was divided in two parts, respectively labeled ‘Reliability’ and ‘Practical usefulness’, to test these hypotheses.

## 2. Materials and Methods 

### 2.1. Participants

Thirteen elite swimmers (five female, eight male) participated in the first part of this study (reliability). All swimmers were members of the Dutch national team. Characteristics of each swimmer are given in Table 1. Swimmers trained approximately 28 h per week, consisting of 20 h of swimming and 8 h of dry land training. Subjects 2, 4, 8 and 9 also participated in the second part of the study (practical usefulness). Their coach was highly experienced and had supervised swimmers during the 2008, 2012 and 2016 Olympic Games, including European, World and Olympic medal winners. The study was approved by the Ethical Committee of the Faculty of Human Movement Sciences, Vrije Universiteit Amsterdam, Amsterdam, The Netherlands. Swimmers were verbally instructed and signed an informed consent form before any measurements were conducted. Written parental consent was obtained for swimmers younger than 18 years of age.

### 2.2. Experimental Set-Up

Measurements took place in an Olympic sized swimming pool during regular training sessions. The first part of the study (see Section 2.2.1) was performed at the regular training environment of the swimmers, the Sloterparkbad, Amsterdam, The Netherlands. The second part was conducted at Thanyapura Sports & Leisure Club (Thalang, Thailand) during a three-week training camp. Water temperature was approximately 27 °C in both swimming pools throughout the testing periods. Moreover, humidity and pressure were similar in both locations since the sensors were under water at all times. Hence, there was no reason to presume that the difference in measurement environment would have an effect on the results.

#### 2.2.1. Video Recordings

**Figure 1:**
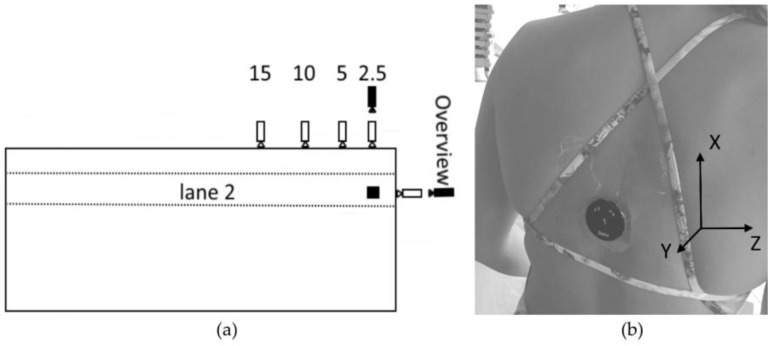
Experimental setup. (**a**) Positioning of the cameras. White cameras are positioned underwater; black cameras are positioned above the water line. (**b**) Sensor location on the upper back secured with film dressing. Picture taken by the researchers with the swimmer’s permission.

The pool in Amsterdam was equipped with seven cameras, five under water (scA1400-30gc, 50 Hz, Basler, Ahrensburg, Germany) and two above the water line (Basler piA640-210gc, 200 Hz). All cameras were synchronized and calibrated for measurements in lane 2, enabling a continuous view of the first 15 m of swimming (see Figure 1a). Synchronization and recording was performed using Streampix (version 5.10.1.0, ×64, Norpix Inc., Montreal, QC, Canada) on an ACP 4360 MB computer (3.20 GHz, 64 bit Windows 7 professional SP 1, Advantech, Taipei, Taiwan). Recordings of each camera were stored in ‘*.seq’ format on a 2 TB hard drive dedicated specifically to the data from that camera. A separate camera (Coolpix AW-120, 25 Hz Nikon, Tokyo, Japan) was placed to obtain an overview of the entire pool. 

#### 2.2.2. Tri-Axial Accelerometers

A Zephyr Bioharness 3 (Annapolis, MD, United States) was used to gather and store (in binary format) acceleration data at a sample frequency of 100 Hz. This sensor consists of a tri-axial accelerometer with a range of from −16 g to +16 g and a 12 bit resolution. It had the following characteristics: dimensions 28 mm × 7 mm, weight 18 grams, operating temperature between −30 and +60 °C and waterproof up to 1 m (IP67). The sensor was placed on the upper back midway between the inferior angle of the left and right scapula using Tegaderm semi-permeable film dressing (10 cm × 12 cm), see Figure 1b. This location was chosen for optimal comfort and minimal drag after pilot testing with regard to user experience. After the measurements the sensors were placed in a unit provided by the manufacturer to be transferred via a USB connection to a computer.

### 2.3. Experimental Protocol

For the first part of the study both the cameras and the accelerometer were used for the determination of stroke type, lap time, stroke count and stroke rate. First, the accelerometer was switched on and fixed to the swimmer’s body at the position described above. Synchronization between the accelerometer and the cameras was ensured by a researcher tapping the accelerometer three times with his right hand clearly visible in front of the 2.5 m above water camera (Figure 1a), a modified version of the routine used by Schoonderwalt et al. [21]. Swimmers then performed a warm-up protocol designed by the coach (Table 2). Video and accelerometer data were collected throughout the warm-up. During this warm-up swimmers swam at a self-selected speed with no requirements on lap times. The warm-up phase was chosen in agreement with the coach. All swimmers performed the same protocol, unlike during regular training. Moreover, from a practical point of view, it was only possible to obtain a maximum of 45 minutes of video recording due to memory restrictions.

The second part of the study was performed during a three-week training camp in January 2016 in preparation for the 2016 Olympic Games. Four athletes, subjects 2, 4, 8 and 9, were measured with accelerometers in the same way as in part 1 during six very similar training sessions. In each session several identical repetitions of one lap were performed with a target lap time (at one decimal) and stroke count set by the coach, indirectly specifying a certain stroke rate. Swimmers were asked to count strokes and to comply with the goals set by their coach. This is something they can do themselves, without any aids. Researchers had no influence on the training program. The results for the six sessions were compared to examine differences between sessions. After discussion with the coach, the fifth session was selected with a prescribed rate of perceived exertion (sRPE) above 8 [22] to examine the compliance within this session. In this session, the swimmers performed four sets of six times 50 m high intensity front-crawl followed by 300 m front-craw pull with paddles and 100 m recovery. As before, the target lap time and stroke count in the six times 50 m were determined by the coach. 

### 2.4. Data Processing

#### 2.4.1. Video data

The video data were manually digitized with custom made software code in Matlab (The Mathworks™, Natick, MA, USA, R2014b, 8.4.0.150421, 64 bit). Several events were digitized: tapping of the accelerometer for synchronization, stroke type of each lap, push-off (last moment of contact with the wall), first moment of contact with the wall of each finish, and entry of the hand for each visible stroke. From these events, the following parameters and characteristics were determined:

- sync time: moment of synchronization between accelerometer and cameras;
- time per 100 m (T100): the time difference between a push-off to the next push-off or finish that was visible in the video data;
- stroke type: front-crawl, backstroke, breaststroke or butterfly;
- stroke count: number of strokes per lap;
- stroke rate: the inverse of the time it takes to complete one stroke (from one entry of the right hand to the next).

An expert operator manually performed the digitization process. The intra-operator reliability was obtained by analyzing the data of two different swimmers twice. TEM, defined as the SD of the difference divided by square root 2 [4], for push-off (n = 24) and entries of the right hand (n = 40) was 0.01 s, while TEM for the finish (n = 12) was 0.02 s. 

#### 2.4.2. Acceleration Data

Acceleration data were processed with custom-made software code in Matlab. In part 1, the same variables were determined from the acceleration data as from the video data: stroke type, T100 (from lap time), stroke count and stroke rate. First the raw data were converted from binary format to acceleration in g (g = 9.81 m·s^−2^), following the specifications provided by the manufacturer. All data processing was based on low-cost methods in order to be able to apply the algorithms in a real-time environment in the future. Signal energy levels (the square of the signal after a high-pass second order Butterworth filter) were calculated for the acceleration in *x*-, *y*- and *z*-direction (a*_x_*, a*_y_* and a*_z_*, see Figure 1b) in accordance with the work of Davey et al. [3]. From these, energy envelopes XE, YE and ZE were made using an exponentially weighted moving average (α = 0.001 [23]). The moving average was used to smooth the signal (with more weight for the most recent values) and obtain clear differences in signal energy in order to distinguish the different stroke types. Also, the resultant acceleration (a*_r_*, using the Euclidean norm) and a corresponding energy envelope (AE) with α = 0.1 were calculated. Separate methods were then used to determine each parameter:

- SyncTime. Detection of three peaks corresponding to the tapping of the accelerometer via manual selection of the maxima.
- Stroke type detection was performed for each data-point using a combination of orientation and energy content of the signal as done by Davey et al. [3], but modified for the Zephyr Bioharness 3. Backstroke was identified by a negative signal along the *y*-axis. For differentiation between front-crawl, butterfly and breaststroke several thresholds were necessary. Similar to Davey et al. [3] the thresholds were based on empirical analysis of large amounts of collected data. When ZE exceeded 0.2 g^2^, data were identified as front-crawl. When XE was above 0.1 g^2^ data were identified as butterfly. Below this threshold but above 0.05 g^2^, data were identified as breaststroke.
- Lap time and T100. The a*_x_* data were low-pass filtered with a second order Butterworth filter of 5 Hz [12]. Subsequently, a numerical gradient of the data along the *x*-axis was used. The resulting signal was squared to determine its energy content. Finally, maxima in the signal energy above 0.02 g^2^ were selected as push-off. To detect finishes, moments of rest and swimming were defined. Swimming was defined to occur when AE was above 0.005 g^2^ (some activity had to take place) and a*_x_* above −0.6 g (when swimmers were almost in a vertical position). Rest was defined to take place when AE and a*_x_* were below these values, i.e., when swimmers were in an almost upright position and showed little activity. The finish was detected when a transition occurred from swimming to rest and both AE and a*_x_* remained below the thresholds for at least a 10-frame window (i.e., 0.1 s). Lap time was the difference between a push-off and the first following push-off or finish larger than 20 s. T100 was the summed lap time of an uneven lane and the following even lane.
- Stroke count and stroke rate. The a*_y_* and a*_z_* data were filtered with a second order Butterworth band-pass filter between 0.25 (to remove gravitational effects) and 0.5 Hz [3,24]. Local maxima were detected when the sum of a*_y_* and a*_z_* exceeded 0.1 *g* and maxima were separated by at least 0.5 s but no more than 4 s, corresponding to stroke rate between 15 and 120 strokes per minute (str·min^−1^). Furthermore, strokes were neglected when detected within 1 second of a push-off. Stroke count was defined as the number of maxima within a lap; stroke rate was defined as the reciprocal of the time difference between two maxima in str·min^−1^.

**Figure 2:**
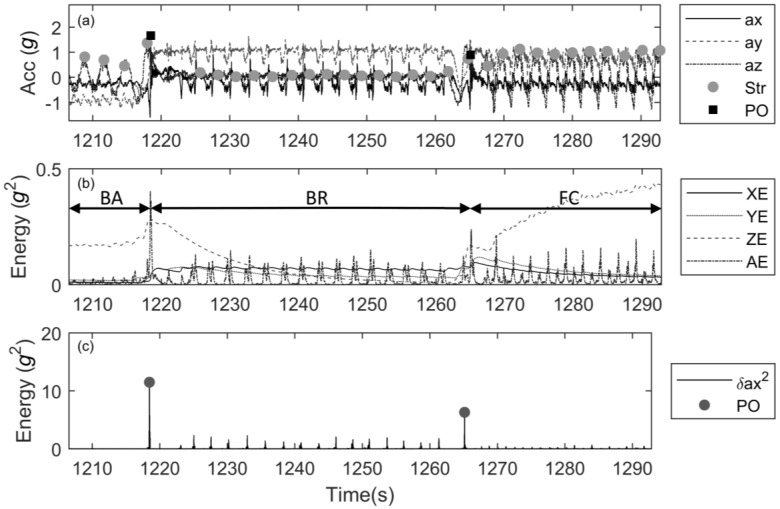
Illustration of the analysis of the acceleration data to obtain lap times, strokes and stroke type. (**a**) raw data with detected strokes (grey dots) and push-offs (black squares). Acceleration (in *g*) in *x*-, *y*- and *z*- direction is shown by a continuous, dashed and dotted line, respectively. A stroke was neglected if it occurred within 0.5 s of a push-off. (**b**) Energy envelope of the signal (in g^2^) in *x*-, *y*- and *z*-direction and resultant acceleration calculated using an exponentially weighted moving average: XE, YE, ZE and AE are shown with a black, grey, dashed and dotted line, respectively. The values are used to detect stroke type. In this example backstroke (BA), breaststroke (BR) and front-crawl (FC) are shown. (**c**) Energy content of the signal (in g^2^) in *x*-direction (EC) from which push-offs (PO, black dots) are detected. Note that detection of push-offs would be more difficult in figure (**b**).

A graphical illustration of the data analysis is shown in Figure 2.

For part 2, lap time, stroke count and stroke rate were determined as in part 1. In addition, another set of parameters was derived. From stroke rate, the mean and SD of stroke rate per lap were calculated and the first stroke within each lap was determined as an indication of the underwater phase duration. Finally, an indication of the stroke index (*SI*) [25] was calculated using the average velocity per lap (*v*) and stroke length (*SL*):

(1) SR=v·SL

*v* was calculated by dividing the pool length through lap time (LT):

(2) v=50/LT

while SL was calculated by dividing *v* through the stroke rate (SR, in Hz):

(3) SL=v/SR

### 2.5. Statistical Analysis

All statistical analyses were conducted in Matlab (The Mathworks™, Natick, MA, USA, R2014b, 8.4.0.150421, 64 bit) unless mentioned otherwise. The data were tested for normality using the Shapiro-Wilks test. The level of significance was set at 0.05. All data are expressed as means with SD within brackets.

For part 1, the reliability of T100, stroke count and stroke rate was identified with the TEM, which was calculated with a spreadsheet downloaded from http://sportsci.org [4] and represented as absolute values. Since calculation of the TEM lacks graphic illustration, Bland-Altman analysis [26] was performed for lap time, stroke count and stroke rate [26] providing the bias and limits of agreement (LoA). 95% confidence intervals are given between brackets. Reliability of stroke type was given as a percentage of the total laps that were determined incorrectly. 

For part 2, descriptive statistics were used, as only 4 subjects were measured. Lap time, stroke count and stroke rate were determined for each lap from all sessions measured. The training output as measured with the accelerometers was compared for each lap with the prescribed training to assess the compliance within a single training. Based on the work of [4] a compliance range of ±2% was defined for lap time and of ±1 stroke for stroke count. Laps were labeled non-compliant (NC) when swimmers performed outside of this range. In addition, a range of ±1 SD from the average mean stroke rate, SD of the stroke rate, under water phase and stroke index was used to identify laps in which subjects performed significantly different for that parameter. To compare the different training sessions a scatter plot was made between lap time and mean stroke rate. 

## 3. Results

The reliability results are presented in Table 3. The average TEM of T100 (0.26 s) was well within the TEM of a stopwatch (0.42 s) [3,4]. Not every finish was perfectly visible on the video footage, because other swimmers were blocking the view or because turbulence or the swimmer’s own body obscured the hand contacting the wall. Stroke type was incorrectly determined for two laps, in which breaststroke was identified as butterfly. This corresponded to 1.28% of all measures.

**Figure 3:**
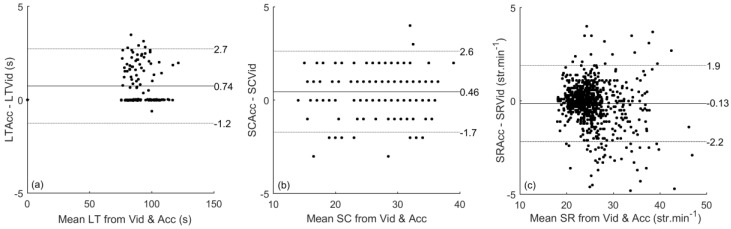
Bland-Altman plots comparing video (Vid) and accelerometer (Acc) data for (**a**) lap time (LT, in seconds), (**b**) stroke count (SC) and (**c**) stroke rate (SR, in strokes per minute). Each dot indicates a data point. Black lines show the mean, while grey lines indicate the limits of agreement.

For the main research parameters, lap time, stroke count and stroke rate a Bland-Altman plot was made (Figure 3). The bias for lap time was 0.74 [0.56 0.91] with LoA from −1.20 [−1.50 −0.90] to 2.70 [2.40 3.00] and CR of 2.00. In Figure 3a two different trends for lap time can be observed, one corresponding to laps ending with a turn and the other to laps corresponding with a finish. As noted in Table 2, push-off detection was more reliable than finish detection. The bias for stroke count was 0.46 [0.32 0.60] with LoA from −1.70 [−1.94 −1.46] to 2.60 [2.36 2.84] and CR of 2.20. The bias for stroke rate was −0.13 [−0.20 −0.06] with LoA from −2.20 [−2.32 −2.08] to 1.90 [1.78 2.02], and CR of 2.00. Figure 3c shows a divergence at higher stroke rate. 

In the second part of the study, subjects reported an sRPE of 9 (SD 0.82) for session 5. A comparison between accelerometer timing and manual timing was not feasible, as the coach was unable to measure all times for all four swimmers during the session. Three of the swimmers were generally compliant and one was generally non-compliant.

**Figure 4:**
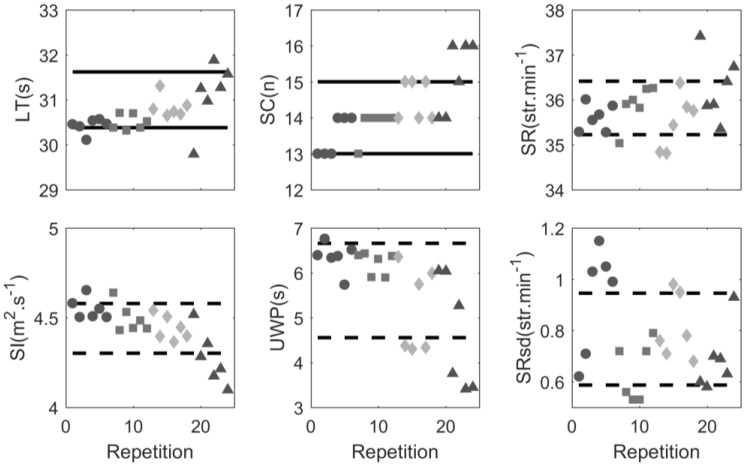
Compliance dashboard for subject 9 in session 5. Top row from left to right: lap time (LT), stroke count (SC), average stroke rate (SR). Bottom row: stroke index (SI), underwater phase (UWP) and standard deviation of stroke rate (SRsd). Each data point represents a lap, with different symbols for different sets: ● = set 1, ■ = set 2, ♦ = set 3, ▲ = set 4. Black lines represent the compliance range. Dotted black lines represent the mean value ±1 standard deviation.

Figure 4 shows an example of a dashboard that was created for one particular subject. The dashboard provides immediate information to a coach or sports scientist. In four laps the subject performed outside of the compliance range for lap time and in three laps outside of the stroke count range. Especially in the final set (indicated by a triangle), the subject had difficulties maintaining compliance. In that set, mean stroke rate, stroke index and under water phase fell out of the ±1 SD range. 

**Figure 5:**
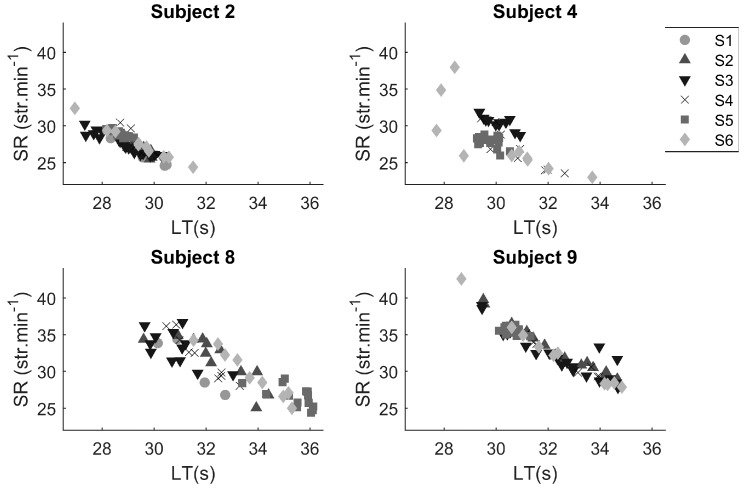
Comparison of different training sessions with lap times (LT) and stroke rate (SR) per subject. Each data point represents a lap, with different symbols for different sessions (S1–S6): ● = session 1, ▲ = session 2, ▼ = session 3, × = session 4, ■ = session 5, ♦ = session 6.

Figure 5 shows the comparison between different training sessions of similar type. Subjects 2 and 8 were measured in all sessions. No data were recorded for subject 9 in session 1 due to malfunction of the sensor, while subject 4 performed training exercises of a different type in sessions 1 and 2. Subjects 2 and 9 showed a relatively constant relationship between lap time and stroke rate over the different training sessions. Subject 8 showed the largest spread. Especially in sessions 5 and 6, a higher stroke rate was attained at a certain lap time. For subject 4, the stroke rate at a certain lap time in session 3 appeared to have been higher than in the other sessions measured. In the last session, subject 4 showed a large variation in lap time and stroke rate—the pacing profile was not constant.

## 4. Discussion

In the first part of this study it was examined whether lap time, stroke count and stroke rate can be detected with high accuracy and reliability using tri-axial accelerometers. The TEMs for the variables of interest were: 0.26 s for lap time, 0.44 for stroke count and 0.72 str∙min^−1^ for stroke rate. With a TEM of 0.57 s the accuracy of the timing of the finish was considerably lower than that of lap time. These results were confirmed by the Bland-Altman plots in Figure 3, although a slight bias was observed for lap time and stroke count. Overall, the results obtained confirmed the first hypothesis that stroke type, lap time, stroke count and stroke rate can be accurately and reliably measured for all stroke types and for multiple swimmers simultaneously using commercially available, water-resistant tri-axial accelerometers. 

In doing so, the present study overcame some of the limitations of previous research; low subject numbers [3,12,16,27], short measurement periods [3,10,11,12,13,14,15,16,17,19,20] and very few studies focusing on all stroke types [12,15,16]. Firstly, we studied the reliability of accelerometers over a longer distance with more participants than previously studied. Secondly, we were able to measure lap time, stroke count and stroke rate for all four stroke types using a finish detection method similar to the one described by Davey et al. [3]. However, even though longer measurement periods were conducted in the present study, it was still not possible to evaluate a complete two hour training session due to the limited memory capacity of the video recording system. Nevertheless, it is likely that the algorithms developed can indeed be used for longer periods of time, since the results were consistent over the entire measurement period. Whether the algorithms used are valid for swimmers of different performance levels could not be determined, because the subjects of the present study formed a very homogeneous group in terms of an invariably high performance level according to FINA points (mean of 858, SD 66) [28]. 

The current results for accuracy and reliability were slightly higher than in previous research. For example, Davey et al. [3] reported an accuracy of 95% for stroke time detection, while we found an accuracy of almost 99%. In contrast, the results for stroke count and stroke rate were similar to those reported in previous studies (TEM < 1). However, we used one algorithm with a combination of the signal in *y*- and *z*-direction for all stroke types, whereas most previous studies focused on front-crawl [3,29] or used separate algorithms for each stroke type [12,30]. It should be noted that there appears to be a larger spread at higher stroke rates (Figure 3c). This is due to the 4 times 50 at the end of the protocol which included technical drills and a build-up from start to finish (see Table 2). Both exercises cause irregularities in the stroke rhythm, which is reflected in the results presented in Figure 3c.

The detection of lap time was more accurate compared to that reported by Davey et al. [3]. Although the off-set was higher (0.74 s versus 0.32 s), the standard deviation was lower (0.37 s versus 0.58 s). This difference might be explained by a different definition of the start of a lane. Davey et al. [3] used a change of orientation as the starting point, whereas we used the last moment of contact with the wall (i.e., the push-off). The push-off is better reproducible than a change of orientation. Unfortunately, finish detection was less reliable than push-off detection (Table 3 and Figure 3). The video recordings showed a large spread in the manner in which a finish was made. Apparently, swimmers finished in different ways (at least during training): some swimmers were in an almost vertical, upright position before finishing, while others remained in supine position several moments after finishing. Furthermore, swimmers sometimes rotated along a longitudinal axis when finishing but not always. This rotation could lead to detection of an extra stroke, which might have caused the bias in stroke count. The finish detection might thus be improved by encouraging swimmers to always make a good finish, i.e., maintain a streamlined position until the wall is touched. The difficulties in finish detection are the main reason for the bias of 0.74 s of lap time detection. The bias of the finish detection was 0.88 s. After compensation for the bias of finish detection, the bias of lap time detection was 0.02 s. Nevertheless, it can be concluded that tri-axial accelerometers can be used to accurately and reliably measure lap time, stroke count, stroke rate and stroke type and we demonstrated that this can be accomplished with low-cost processing methods that allow usage of the algorithms in real-time when such is possible. Further improvements can be obtained by using information from additional sensors, such as gyroscopes [17,31]. 

The few validation trials found in literature [18,32], often used a short testing protocol with a limited number of laps [3,12,13,33]. In the current research 1200 m of swimming was measured in a group of 13 swimmers. The high reliability and accuracy over a longer testing protocol are promising outcomes for training practice. As described in the preceding, coaches currently use a stopwatch to measure training output, usually timing various characteristics of several swimmers simultaneously. This may lead to an especially complicated and hectic situation when swimmers are starting and finishing on both sides of the pool, in different lanes and with different training programs per individual. While the accuracy and reliability of the measurements by the coach rapidly declines when more swimmers are present, the results from the tri-axial accelerometer algorithms will remain the same and thus will be superior to manual timing. As such, the results of part 1 of the present research were the input requirements of part 2. 

The results of part 2 demonstrate that the evaluation of training compliance is possible using tri-axial accelerometers in a group of elite swimmers (n = 4). Compliance, defined as the fulfillment of the training aims as defined by the coach, was assessed both within and between different training sessions. A large spread was found in the relationship between lap time and stroke rate over different sessions with similar training aims. Hence, also the second hypothesis that tri-axial accelerometers can be used to determine the compliance of elite swimmers to the training program prescribed by the coach, enabling fast and accurate feedback on multiple parameters simultaneously was confirmed. However, we were unable to study if higher compliance leads to better performance outcomes at the important competitions of the year, because the number of participants and training sessions studied was too small for this purpose.

The compliance boundaries of the dashboard (Figure 4) were based on the TEM of lap time and stroke count and on SD of mean stroke rate, variation of stroke rate, stroke index and under water phase. These boundaries were used based on the work of Hopkins [4]. However, it remains unclear if this is the optimal range for individual athletes in sports practice. The method does provide more detailed insight into what happens with a certain swimmer when maintaining lap time or stroke count is difficult within a session. The fact that swimmers applied different solutions to cope with the task highlights the necessity of individual monitoring of elite athletes [34]. The dashboard (Figure 4) shows how stable the pacing profile is and provides the coach with more detailed information about individual training compliance, information that the coach otherwise would not have, except for the lap times. This information may lead to a different focus in future training sessions. For instance, subject 9 might focus on maintaining a long under water phase to comply with a certain lap time and stroke count. In addition, she swam the fastest lap in the first repetition of set 4. It appears she spent too much energy, since stroke rate was high while she was unable to maintain the prescribed lap time. The coach might tell this swimmer to control herself in the first repetition of a set in order to maintain compliance.

To our knowledge, only Stewart and Hopkins [1] studied the compliance of exercise performance with exercise prescription in swimming. They found that swimmers were able to perform the prescribed distances and to stick to the rest intervals, but were less able to comply with the prescribed velocity pace especially on an individual level as indicated by a poor relationship between prescribed and observed velocity pace (*r* = 0.30). In the current research, the coach prescribed both lap time and stroke count. Unfortunately, it is difficult to compare the results of this session with the work of Stewart and Hopkins [1], since only one velocity (via lap time) was set in our study and not a range of velocities. 

The potential of repetitive measurements is evident from Figure 5. With this information, training sessions can be designed with new targets for lap time and stroke count. Subject 2 and 9 showed a small variation around the relationship between lap time and stroke rate over different training sessions, whereas subjects 4 and 8 showed much more variation. The question remains why this variation occurred, how much variation is preferable or inevitable and how a coach should treat this variation. It is unlikely that performance level explains the variation [35], because the subjects were all elite swimmers (see Table 1). More likely, the variation might have been due to the complexity of the task at hand, which involved an additional constraint: swimmers had to swim a certain lap time, with a certain stroke count. This constraint is likely to have an effect on the coordination and the variability of the stroke pattern of the swimmer [36]. Furthermore, the task was difficult from a physiological point of view. The swimmers fatigued, which is known to lead to more variability [37]. Indeed, fatigue leads to less efficient swimming [38]. This might be reflected in physiological parameters such as heart rate [39] and/or in changes in intra-cyclic acceleration [40]. Future research could include heart rate for which an additional monitor would be necessary, but it should be possible to measure intra-cyclic acceleration with the current set-up. This will lead to interesting new areas of investigation for future research with direct relevance for training practice. 

## 5. Conclusions

Tri-axial accelerometers can be used to accurately and reliably measure swimming parameters and monitor training performance. Tri-axial accelerometers may thus be used to examine the compliance of individual swimmers to preset training targets. Moreover, they provide the coach with more, and more detailed, information to guide and control individual training programs. When real-time processing and transmission of the relevant data become reality, coaches will no longer have to use a stopwatch to monitor the training of multiple swimmers. As a result, they will have more time, for instance, to provide swimmers with feedback on technique. Continuous monitoring of the training process with tri-axial accelerometers might offer additional information on dose-response relationships and the balance between load and load capacity. This makes tri-axial accelerometers a powerful tool for coaches to optimize the training program.

## Figures and Tables

**Table 1:** Subject characteristics presented in descending order of FINA points. M = Male, F = female, BU = butterfly, FC = front-crawl, BA = backstroke, BR = breaststroke. FINA points were obtained from www.swimrankings.net (12 September 2016). Four of the subjects also participated in part 2 of the study, as indicated in the last column.

Subject	Gender (M/F)	Age	Height (cm)	Weight (kg)	Experience (Years)	Stroke	Highest FINA Points	Part 1	Part 2
1	F	29	182	66	24	BU, FC	974	Yes	No
2	M	26	193	85	21	FC	940	Yes	Yes
3	M	20	190	80	14	BA	881	Yes	No
4	M	27	203	93	20	FC	869	Yes	Yes
5	M	25	190	82	13	BR	865	Yes	No
6	F	18	179	62	14	FC	861	Yes	No
7	M	19	183	73	12	BR	853	Yes	No
8	M	18	198	98	9	BU	841	Yes	Yes
9	F	17	172	61	9	FC	803	Yes	Yes
10	F	17	180	72	8	FC	798	Yes	No
11	F	17	177	60	10	BA	788	Yes	No
12	M	19	195	85	10	BU, FC	779	Yes	No
13	M	17	185	76	7	FC	690	Yes	No
Mean		21	187	76	13		858		
SD		4	9	12	5		66		

**Table 2:** The warm-up protocol performed in part 1 of the study. FC = front-crawl, BA = backstroke, BR = breaststroke, BU = butterfly.

Repetitions	Distance	Task	Rest
1	400	100 FC, 50 BA, 50 BR, 100 FC, 50 BA, 50 BU	20 s
1	300	50 kick, 100 pull, 50 kick, 100 pull	20 s
1	200	Individual Medley	20 s
1	100	Pull	20 s
4	50	Uneven: technical drills. Even: Build-up from start to finish	20 s
Total	1200		

**Table 3:** Group differences between accelerometer and video analyses and reliability of the investigated parameters. SR = stroke rate, PO = push-off, F = finish, T100 = time per 100 m, SC = stroke count, ST = stroke type, TEM = typical error of measurement, CI = 95% confidence intervals, n = number of observations.

Parameter	Difference	TEM (CI)	n
PO (s)	0.01	0.03 (0.02–0.03)	111
F (s)	0.88	0.57 (0.49–0.69)	65
T100 (s)	0.74	0.26 (0.23–0.29)	132
SC	0.44	0.73 (0.67–0.81)	235
SR (str∙min^−1^)	0.05	0.72 (0.69–0.75)	888
ST (%)	1.28	-	289
